# Characterization of Volatile Organic Compounds of Healthy and Huanglongbing-Infected Navel Orange and Pomelo Leaves by HS-GC-IMS

**DOI:** 10.3390/molecules25184119

**Published:** 2020-09-09

**Authors:** Shan Cao, Jingyu Sun, Xiaoyong Yuan, Weihui Deng, Balian Zhong, Jiong Chun

**Affiliations:** National Navel Orange Engineering Research Center, College of Life Sciences, Gannan Normal University, Ganzhou 341000, China; scoral29116@163.com (S.C.); SJYnj_1997@163.com (J.S.); gzyuanxiaoyong@163.com (X.Y.); dwh110by@163.com (W.D.); bal.zh@163.com (B.Z.)

**Keywords:** Huanglongbing, navel orange, pomelo, leaf volatiles, HS-GC-IMS, principal component analysis (PCA)

## Abstract

The Asian citrus psyllid (ACP), *Diaphorina citri* Kuwayama, is the only natural vector of bacteria responsible for Huanglongbing (HLB), a worldwide destructive disease of citrus. ACP reproduces and develops only on the young leaves of its rutaceous host plants. Olfactory stimuli emitted by young leaves may play an important role in ACP control and HLB detection. In this study, volatile organic compounds (VOCs) from healthy and HLB-infected young leaves of navel orange and pomelo were analyzed by headspace-gas chromatography-ion mobility spectrometry (HS-GC-IMS). A total of 36 compounds (including dimers or polymers) were identified and quantified from orange and 10 from pomelo leaves. Some compounds showed significant differences in signal intensity between healthy and HLB-infected leaves and may constitute possible indicators for HLB infection. Principal component analysis (PCA) clearly discriminated healthy and HLB-infected leaves in both orange and pomelo. HS-GC-IMS was an effective method to identify VOCs from leaves. This study may help develop new methods for detection of HLB or find new attractants or repellents of ACP for prevention of HLB.

## 1. Introduction

Huanglongbing (HLB), also known as citrus greening disease, is a worldwide destructive disease of citrus [1]. HLB has caused several billion dollars in losses to the citrus industry in Florida, USA: Citrus-bearing acres have decreased from 679,000 in 2003–04 to 402,000 in 2017–18 and the number of citrus growers went down from 7389 in 2002 to 2775 in 2017 [2]. HLB-infected citrus trees have yielded fewer and poorer-quality fruits, which are less juicy, and bitter and metallic in taste [3,4]. The Asian citrus psyllid (ACP), *Diaphorina citri* Kuwayama, is the only vector of bacteria responsible for HLB [5]. Novel and sustainable approaches to the control of ACP are urgently needed for successful HLB management programs. 

ACP mates, oviposits, and develops exclusively on new flush shoots [6]. Recent studies have shown that volatile organic compounds (VOCs) emitted by flushing shoots may play an important role in the detection, location, and evaluation of potential host plants by ACP [7]. The ability to understand the chemical composition of citrus leaf VOCs may facilitate ACP’s ability to recognize the stimuli signal from its host plant and the interaction between them. Wenninger et al. demonstrated that ACP used olfactory cues in orientation to host plants and suggested using plant VOCs to monitor and manage ACP [8]. Robbins et al. used GC-MS to identify compounds in headspace volatiles collected from uninfected flush and young leaves of various citrus genotypes [9]. Alquézar et al. found that (*E*)-β-caryophyllene, a VOC from guava, exerts a repellent effect on ACP [10]. Andrade et al. studied the chemical composition of volatile oils from 22 genotypes of citrus and related genera and speculated that phytol, (*Z*)-β-ocimene, and β-elemene may act as repellents to ACP [11].

In spite of finding efficient repellents or attractants to control ACP, detection of HLB is also very important. Currently, polymerase chain reaction (PCR) is widely used for HLB detection [12,13]. However, this technique has limitations because of the low concentration of bacteria in the infected trees, and HLB symptoms may not appear on leaves for a long time after infection [14]. An alternative approach for detection of HLB is monitoring the VOCs emitted by the plant, which may serve as an indicator to discriminate healthy and infected trees. Olfactory cues have been shown to be important in HLB detection. Aksenov et al. illustrated that changes in VOCs, including linalool, tetradecane, and phenylacetaldehyde, were correlated with HLB-infected trees at the asymptomatic stage [15]. Wang et al. reported gas biosensor arrays for the detection of VOCs released by HLB-infected citrus trees [16]. The typical techniques for VOC detection utilize gas chromatography (GC) in combination with various detectors, including flame ionization (GC-FID), mass spectrometry (GC-MS), and differential mobility spectrometry (GC-DMS) [17]. 

China is one of the world’s leading producers of citrus [18]. The Newhall navel orange industry in Gannan of China covers an area of 0.29 million acres with a total annual output of more than one million tons of oranges. Pomelo (*Citrus maxima* (Burm.) Merr.) is also a popular citrus variety cultivated in Southern China with an annual yield around 0.5 million tons [19]. HLB is the number one threat and responsible for major economic loss to citrus production in China. Monitoring plant health and detecting infection are important to reducing HLB spread and facilitating effective management practices. There are many methods and devices that have been reported to extract and analyze VOCs in plant leaves, such as simultaneous distillation–extraction (SDE) [20], steam distillation (SD) [21], and supercritical fluid extraction (SFE) [22]. Wang et al. analyzed the components of volatiles from the new shoots of six healthy host plant species via the headspace solid-phase microextraction (HS-SPME) method and showed that β-caryophyllene was the characteristic VOC in flushing shoots of the tested ACP host plant species [23]. However, they did not analyze VOCs from HLB-infected species. Sharma et al. used a portable GC device for rapid, in situ, dynamic monitoring of the VOCs produced by milkweeds under aphid attack [24]. New technologies with greater reliability, precision, and accuracy are needed to detect HLB and control ACP. In our study, young leaves of two varieties of citrus, Gannan Newhall navel orange and Shatian pomelo (*Citrus maxima* (Burm.) Merr. cv. Shatian Yu): Healthy orange leaf (HEAO), Huanglongbing-infected orange leaf (HLBO), healthy pomelo leaf (HEAP), and Huanglongbing-infected pomelo leaf (HLBP), were chosen as studying samples. The headspace-gas chromatography ion mobility spectrometry (HS-GC-IMS) technique was used to identify the composition difference between healthy and HLB-infected leaves.

Ion mobility spectrometry (IMS) is a powerful and accurate analytical technique with high sensitivity and simplicity, which hardly requires sample preparation steps [25]. It can detect and characterize chemical substances based on the different migration rates of gas-phase ions under an electric field. The IMS technique has been employed widely and successfully to detect chemicals in agricultural analysis, foods, and pharmaceutics [26]. It has been proved to be a very effective technique for the identification and quantification of VOCs with different properties in the gas phase [27]. Combining IMS with GC is a more efficient method to make better use of its advantages, especially improving the analytical selectivity of complex samples [28]. Headspace-gas chromatography ion mobility spectrometry (HS-GC-IMS) is the technique that combines GC-IMS with headspace sampling. It could be used to characterize VOCs in different samples with low detection limits and good selectivity [29,30]. 

In this study, we conducted a preliminary investigation of VOCs emitted by the young leaves of navel orange and Shatian pomelo, which are representative ACP host plants. The volatile fingerprints of healthy orange leaf (HEAO), pomelo leaf (HEAP), Huanglongbing-infected orange leaf (HLBO), and pomelo leaf (HLBP) samples were established by HS-GC-IMS. The use of HS-GC-IMS to analyze VOCs in orange and pomelo leaf samples and distinguish healthy and HLB-infected leaves has not been reported. This work might provide a reference to develop a new method for detection of HLB and find new attractants or repellents of ACP for prevention of HLB.

## 2. Results and Discussion

### 2.1. HS-GC-IMS Topographic Plots of HEAO, HLBO, HEAP, and HLBP

**Figure 1:**
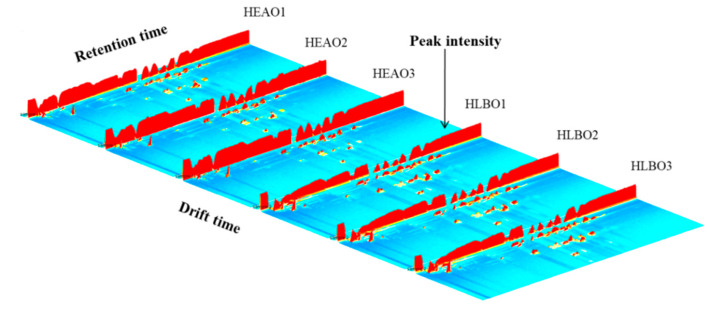
Three-dimensional topographic images of VOCs from healthy and Huanglongbing (HLB)-infected orange leaves (healthy orange leaf (HEAO) and Huanglongbing-infected orange leaf (HLBO)). Number 1, 2, 3 indicate triplicate experiments.

**Figure 2:**
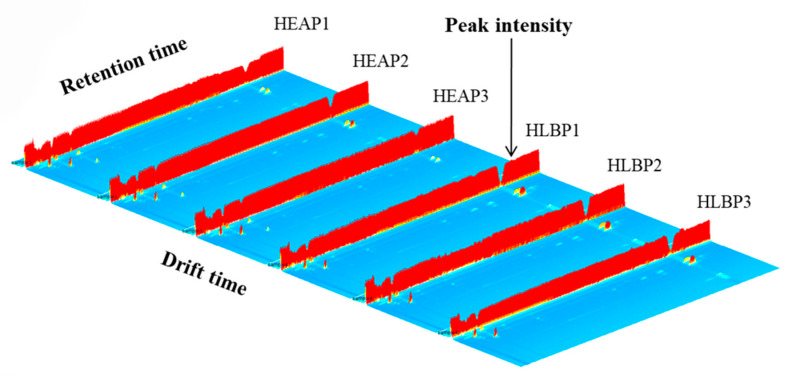
Three-dimensional topographic images of VOCs from healthy and HLB-infected pomelo leaves (healthy pomelo leaf (HEAP) and Huanglongbing-infected pomelo leaf (HLBP)). Number 1, 2, 3 indicate triplicate experiments.

The information of VOCs of HEAO, HLBO, HEAP, and HLBP were obtained via HS-GC-IMS analysis. A 3D spectrum was generated by a Flavor Spec^®^ instrument, as shown in Figure 1 and Figure 2. The X-axis denotes the ion drift time, the Y-axis denotes the retention time of the gas chromatograph, and the Z-axis denotes the peak intensity in the topographic map. The VOCs in different samples demonstrated varying peak intensities. Number 1, 2, 3 indicate triplicate experiments, for example, HEAO1, HEAO2, and HEAO3 were triplicate experiments of HEAO. HLBO had more peak signals of VOCs than HEAO, and HLBP had more peak signals of VOCs than HEAP. A study on the changes of metabolites in citrus leaves in response to ACP stress might be helpful for HLB detection and ACP control.

**Figure 3:**
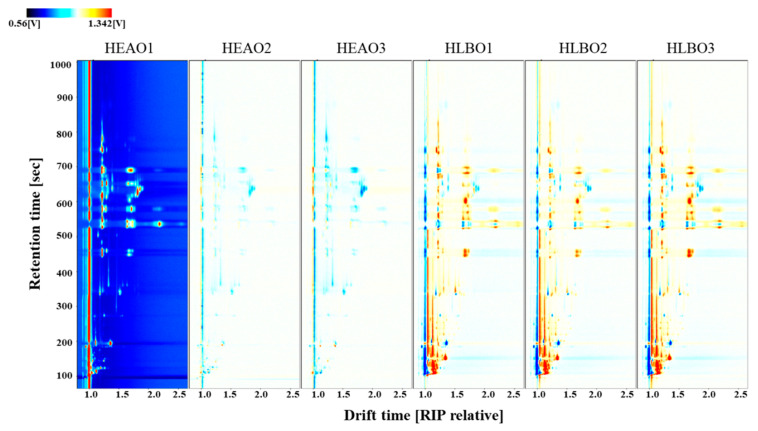
Comparison of ion migration chromatogram of volatile organic compounds from healthy and HLB-infected orange leaves (HEAO and HLBO).

For the convenience of comparison, a vertical view was used as shown in Figure 3. The background of HEAO1 is blue, and the red vertical line at horizontal coordinate 1.0 is the reactant ion peak (RIP, normalized drift time of 7.93). Each point on the right side of RIP represents a VOC. The spectral diagram of HEAO1 was selected as the reference, while the spectral diagram of other samples was deducted from the reference. If two VOCs were identical, the background after deduction would be white. Peak intensities are indicated by different colors. Red spots indicate a higher concentration of the VOCs than the reference, whereas blue spots indicate a lower concentration of the VOCs. The data were displayed at the topographic plot zone with a retention time from 100 to 1000 s and drift time (RIP relative) from 1.0 to 2.5. It is obviously shown that HLBO had more VOC peak signals, and most VOCs had a higher concentration than HEAO.

**Figure 4:**
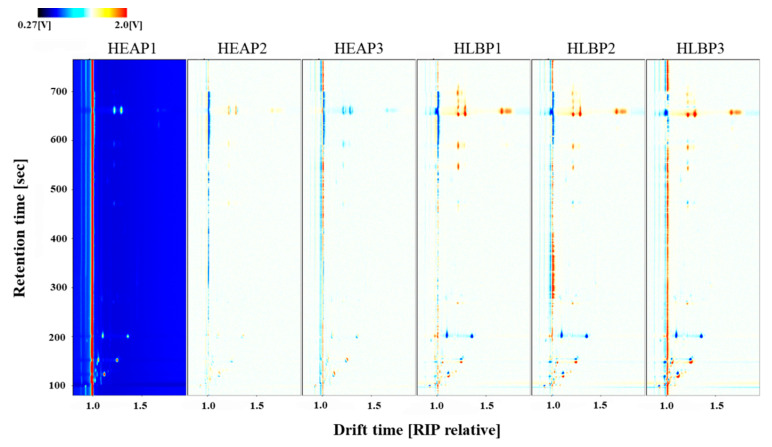
Comparison of ion migration chromatogram of volatile organic compounds from healthy and HLB-infected pomelo leaves (HEAP and HLBP).

A comparison of volatile organic compounds from HEAP and HLBP is shown in Figure 4. It is obviously shown that HLBP had more VOC peak signals and most VOCs had a higher concentration than HEAP.

### 2.2. Differences in the Characteristic Volatile Fingerprints of HEAO, HLBO, HEAP, and HLBP

**Figure 5:**
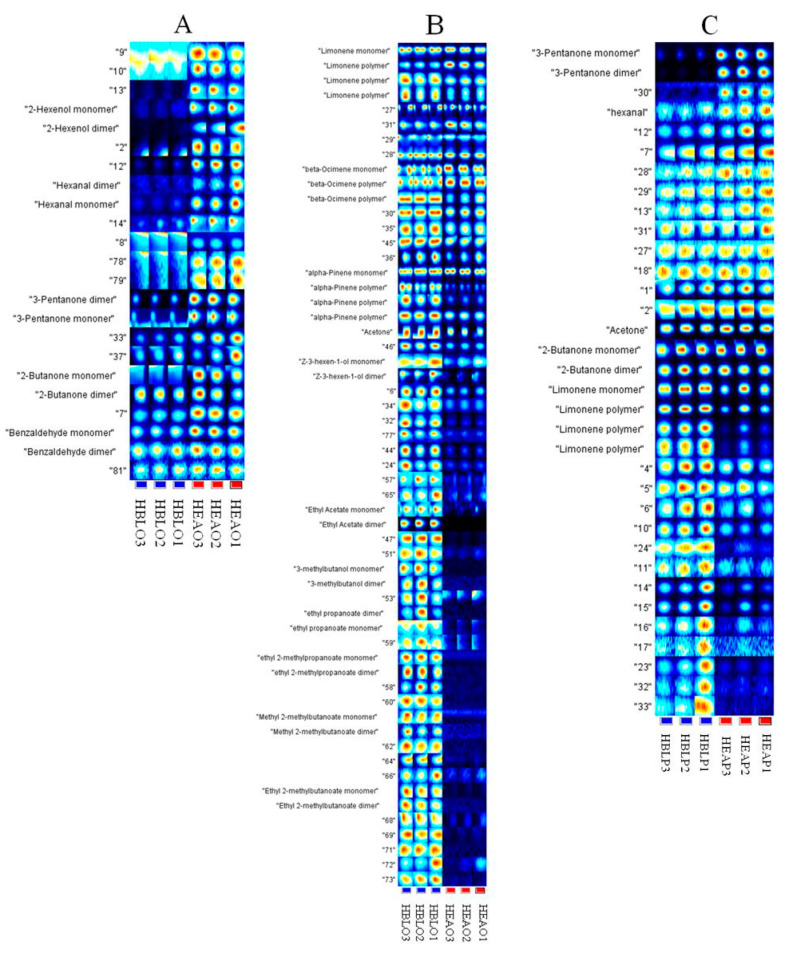
Two parts (**A**,**B**) of the fingerprints of HEAO and HLBO samples and the full fingerprints (part (**C**)) of HEAP and HLBP generated using the Gallery.

Based on the peak signal of the topographic plots, the fingerprints of HEAO and HLBO were generated using the Gallery Plot to accurately evaluate the VOCs, as shown in Figure 5. The full fingerprint of VOCs from the orange leaves HEAO and HLBO was divided into two parts as A and B for better comparison. The full fingerprint of VOCs from the pomelo leaves HEAP and HLBP is presented in part C.

In the fingerprint, each row represents the entire signal peak of one sample, and each column represents the same VOC in different samples. The content of VOCs is distinguished by colors. The higher the content, the brighter the color. VOCs with the same name in the fingerprints are presented as monomers, dimers, or polymers. The drift time of dimers or polymers was increased due to their proton affinity and higher content [27]. The composition and contents of VOCs in HEAO and HLBO can be compared intuitively using fingerprints. Unfortunately, some VOCs were not identified, due to the limited data library. A whole VOC profile should be seen using GC-MS data and HS-GC-IMS data together.

As shown in Figure 5A, ten peaks, including hexanal, 3-pentanone, and 2-butanone, were identified. The brightness of the fingerprint in part A was much stronger in the HEAO fingerprint than that of HLBO. The numbers of identified VOCs in HEAO were more than those in HLBO. Some VOCs, such as 2-hexanol and its dimer, appeared in HEAO, while their fingerprint information in HLBO was minimal. In addition, 3-pentanone was present in HLBO; however, the brightness of its fingerprint was much weaker than that in HEAO. As shown in Figure 5B, twenty-six peaks, including terpenes (limonene, α-pinene, and β-ocimene) and other VOCs were identified. Most peaks in Part B showed a much brighter fingerprint in HLBO than that of in HEAO.

The fingerprints of HEAP and HLBP were generated using the Gallery Plot to accurately evaluate the VOCs in pomelo leaves, as shown in Figure 5C. Ten peaks, including hexanal, 3-pentanone, 2-butanone, and limonene, were identified. Most peaks showed a much brighter fingerprint in HLBP than that in HEAP.

### 2.3. Identification of Volatile Organic Compounds in HEAO, HLBO, HEAP, and HLBP

**Figure 6:**
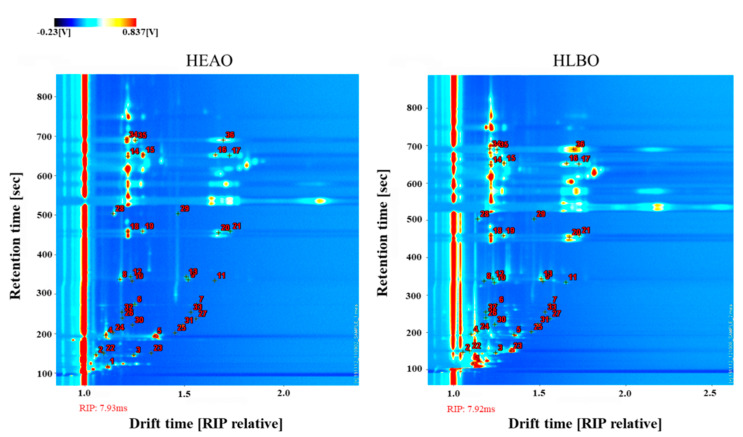
Two-dimensional topographic plots of VOCs in HEAO and HLBO obtained at the retention time and the normalized drift time by HS-GC-IMS. Each marked dot represents a type of identified VOC with the same serial number presented in Table 1.

The qualitative analysis of VOCs in HEAO and HLBO is represented in Table 1 and Figure 6. Some VOCs presented multiple signals as monomers, dimers, and polymers, due to their varying concentrations and adducts formation while moving through the IMS drift tube [27]. These VOCs had the same GC retention times, but different drift times. Table 1 lists all the identified VOCs from the GC-IMS library in orange leaf samples, including the compound name, retention index (RI), retention time (Rt), drift time (Dt) (RIP relative), and signal intensity (SI). RI values were calculated using the homologous series of *n*-2-ketones C4-C9: 2-butanone, 2-pentanone, 2-hexanone, 2-heptanone, 2-octanone, and 2-nonanone, as external standard on the FS-SE-54-CB capillary column. Acetone should be excluded for further analysis because it might come from the cleaning agent. As shown in Figure 6, the two-dimensional topographic plots of VOCs in HEAO and HLBO were obtained at the retention time and the normalized drift time by HS-GC-IMS. Each marked dot represents a type of identified VOC with the same serial number presented in Table 1. The higher the intensity of the red color, the higher the concentration of VOCs; the blue color has the opposite interpretation. These plots show some visual differences of VOCs by location and relative content between healthy and HLB-infected orange leaves.

Highly significant differences (*p* < 0.001) in signal intensity between HEAO and HLBO were observed for 3-pentanone and its dimer, ethyl 2-methylbutanoate, limonene, α-pinene, ethyl acetate and its dimer, ethyl 2-methylpropanoate, benzaldehyde, and methyl 2-methylbutanoate. For each compound identified, the percent difference of average signal intensity between HEAO and HLBO samples was compared (Table 1). The following equation for percent difference was utilized:

Difference = [(SI_HAEO_ − SI_HLBO_)/(SI_HAEO_)] × 100% (1)

**Figure 7:**
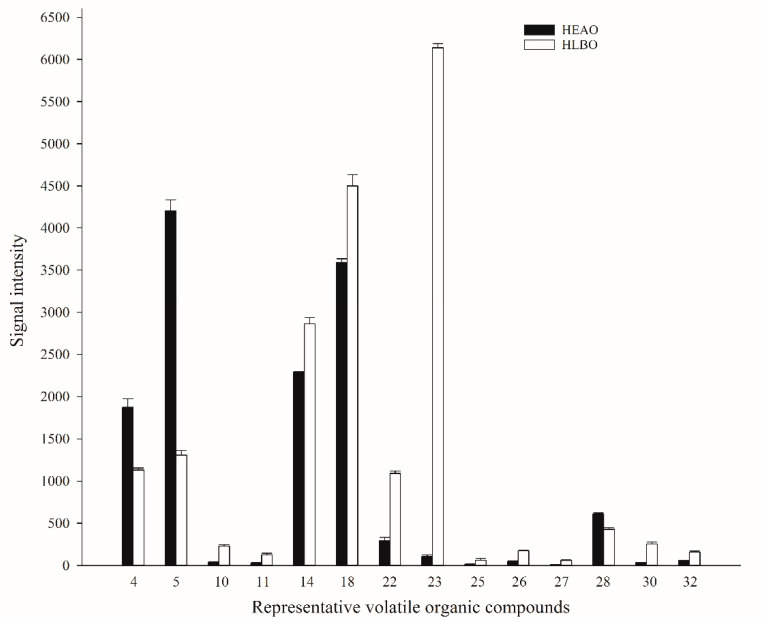
Representative VOCs showing highly significant difference (*p* < 0.001) or the largest percent difference (>300%) of signal intensity in healthy and HLB-infected leaves. The numbers on the Y-axis denote the same compound numbers in Table 1.

The largest percent differences (higher than 300%) were for ethyl acetate dimer (5733.5%), 3-methylbutanol (684.9%), ethyl 2-methylbutanoate (611.6%), ethyl 2-methylpropanoate dimer (402.8%), ethyl 2-methylbutanoate dimer (380.3%), and ethyl propanoate dimer (317.6%). Of the compounds for which there was a highly significant difference, the HEAO signal intensity was higher for 3-pentanone, 3-pentanone dimer, and benzaldehyde. Conversely, the HLBO signal intensity was higher for ethyl 2-methylbutanoate, limonene, α-pinene, ethyl acetate and its dimer, ethyl 2-methylpropanoate, and methyl 2-methylbutanoate. VOCs showing a significant difference in signal intensity in both leaves might be possible indicators for detection of HLB. Representative VOCs showing a highly significant difference (*P* < 0.001) or the largest percent difference (>300%) of signal intensity in healthy and HLB-infected leaves are shown in Figure 7.

**Figure 8:**
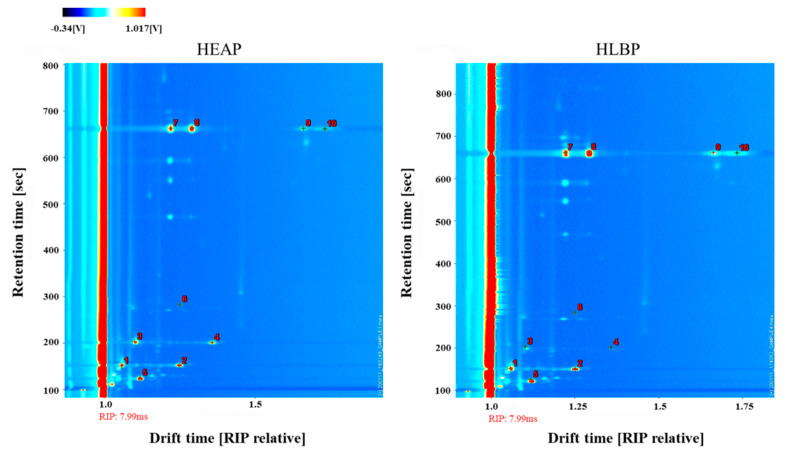
Two-dimensional topographic plots of VOCs in HEAP and HLBP obtained at the retention time and the normalized drift time by HS-GC-IMS. Each marked dot represents a type of identified VOC with the same serial numbers presented in Table 2.

The qualitative analysis of VOCs in HEAP and HLBP is represented in Table 2 and Figure 8. Each marked dot in the two-dimensional topographic plot in Figure 8 represents a type of identified VOC with the same serial number presented in Table 2. VOC content was determined by the brightness degree of color. These plots showed some visual differences in VOCs by location and relative content between healthy and HLB-infected pomelo leaves. The number of identified characteristic peaks (nine characteristic peaks excluding acetone) from the GC-IMS library in pomelo leaf samples was less than that of orange leaf (35 characteristic peaks excluding acetone). The signal intensity of 3-pentanone, 3-pentanone dimer, and limonene polymer has shown a highly significant difference (*p* < 0.001) between healthy and HLB-infected Shatian pomelo leaves. However, the signal intensity of 2-butanone, 2-butanone dimer, and hexanal did not show a significant difference between HEAP and HLBP. For each compound identified, the percent difference of average signal intensity between HEAP and HLBP samples was compared. The following equation was used for percent difference:

Difference = [(SI_HAEP_ − SI_HLBP_)/(SI_HAEP_)] × 100% (2)

The largest percent differences (higher than 200%) were for limonene polymer (283.7% and 239.7%). These differences could be visually compared in Figure 8 where compounds **9** and **10**, which represent limonene polymer in the plot of HLBP, had a brighter color than compounds **9** and **10** in the plot of HEAP. The differences between healthy and HLB-infected Shatian pomelo leaves might provide information for possible indicators for detection of HLB.

### 2.4. Similarity Analysis of Fingerprint Based on PCA

**Figure 9:**
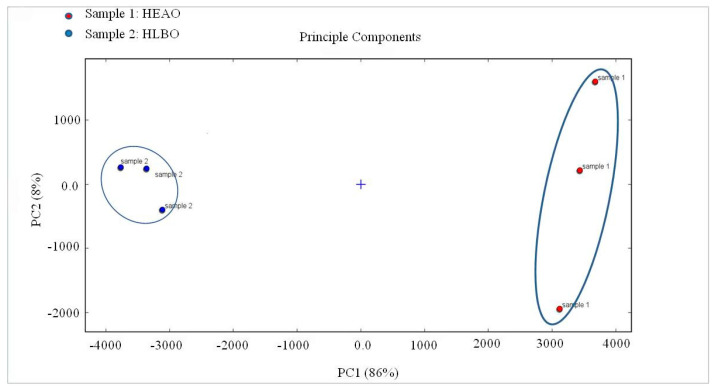
Principal component analysis (PCA) of HEAO (sample 1) and HLBO (sample 2).

Principal component analysis (PCA) is a multivariate statistical analysis technique. By determining a few principal component factors to represent many complex variables in the samples, the regularity and difference among samples could be evaluated according to the contribution of principal component factors [30]. PCA was established using signal intensity to highlight the differences of VOCs in HEAO and HLBO samples, as shown in Figure 9. The distribution map for the first two principal components determined by PCA is displayed, which describes 86% and 8% of the accumulative variance contribution rate, and a visualization map was obtained. The PCA results clearly show that HEAO (sample 1) and HLBO (sample 2) in a completely independent space would be well-distinguished in the visualization map. HEAO could be well-distinguished according to the positive score values of PC1, while HLBO could be well-defined according to the negative scores of PC1, and the difference in HEAO and HLBO could be distinguished by combining with the score values of PC2.

**Figure 10:**
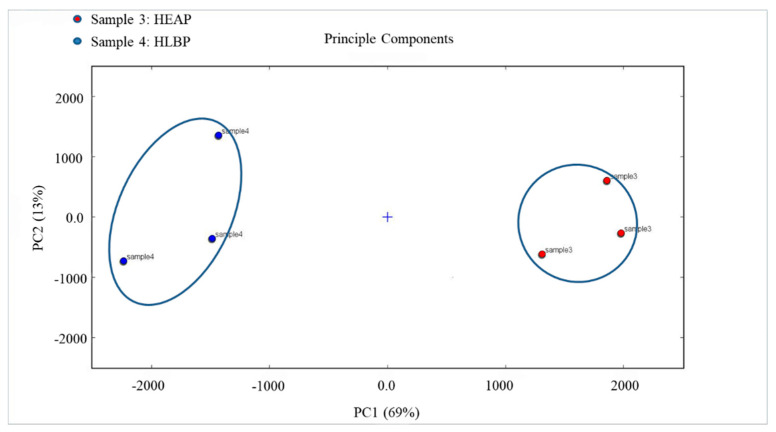
Principal component analysis (PCA) of HEAP (sample 3) and HLBP (sample 4).

PCA of the VOCs in HEAP and HLBP samples is shown in Figure 10. The distribution map for the first two principal components is displayed, which describes 69% and 13% of the accumulative variance contribution rate. The PCA results clearly show that HEAP (sample 3) and HLBP (sample 4) in a completely independent space would be well-distinguished in the visualization map. HEAP could be well-distinguished according to the positive score values of PC1, while HLBP could be well-defined according to the negative scores of PC1, and the difference in HEAP and HLBP could be distinguished by combining with the score values of PC2.

## 3. Materials and Methods

### 3.1. Materials

Gannan Newhall navel orange and Shatian pomelo (*Citrus maxima* (Burm.) Merr. cv. Shatian Yu) young leaves were used as the experiment material and collected in November 2019 and January 2020, respectively, from the orchard of Gannan Normal University, Ganzhou City in Jiangxi Province, China. The HLB-infected leaves were tested by the polymerase chain reaction (PCR) method. 

### 3.2. GC-IMS Analyses

#### 3.2.1. Apparatuses

Analyses of samples were completed on a combined device of an Agilent 490 gas chromatograph (Agilent Technologies, Palo Alto, CA, USA) using a FS-SE-54-CB capillary column (15 m × 0.53 mm), and an IMS instrument Flavor Spec^®^ (Gesellschaft für Analytische Sensorsysteme mbH, Dortmund, Germany), equipped with an autosampler unit (CTC Analytics AG, Zwingen, Switzerland), was used in this study.

#### 3.2.2. HS-GC-IMS Analysis Methods

The analysis method was performed as described by Yang et al. [27]. Fresh leaf (1 g, without any pretreatment) was cut into small pieces and transferred to a 20 mL headspace vial and then incubated at 40 °C for 20 min. Then, a 200 μL headspace was injected into the heated injector using a syringe at 85 °C. Nitrogen (99.99% purity) was used as the carrier gas. The sample was driven into an FS-SE-54-CB capillary column (15 m × 0.53 mm) by nitrogen at the following programmed flow: 2 mL/min for 2 min, 10 mL/min for 10 min, 100 mL/min for 10 min, and 150 mL/min for 30 min. The analytes were separated at 40 °C in the column and then ionized in the IMS ionization chamber at 45 °C. Drift gas flow was set at a constant flow of 150 mL/min. All analyses were performed in triplicate. VOCs were identified by comparing retention index (RI) and the drift time (the time taken for ions to reach the collector through the drift tube, in milliseconds) standard in the GC-IMS library (Gesellschaft für Analytische Sensorsysteme mbH, Dortmund, Germany).

### 3.3. Statistical Analysis

The analytical software included a Laboratory Analytical Viewer (LAV, Dortmund, Germany), three plug-ins (G.A.S. Dortmund, Germany), and a GC-IMS library search. IMS data were acquired and processed using LAV processing software and used to generate the analytical spectrum, where each point represented a VOC. The spectrogram differences were compared using the Reporter plug-in. The differences of fingerprint in different samples were compared via the Gallery Plot plug-in. Qualitative analysis of VOCs was achieved based on the National Institute of Standards and Technology (NIST) and IMS databases from the software’s built-in GC-IMS library. Statistical analyses of the differences between mean values obtained for experimental groups were calculated using IBM SPSS Statistics 23.0. (IBM Corp. Released 2015. IBM SPSS Statistics for Windows, Version 23.0. Armonk, NY, USA). *p* values were calculated using a *t*-test between healthy and HLB-infected leaves for each compound. *p* values < 0.05 were regarded as significant, *p* values < 0.01 as very significant, and *p* values < 0.001 as highly significant.

## 4. Conclusions

As plant leaves are a major source of VOCs emitted in the atmosphere and plant foliar VOCs are very important in mediating plant–plant and plant–insect communication, many methods and analytical techniques have been developed for plant foliar VOC research [31]. Comparison of VOCs in navel orange and pomelo healthy and HLB-infected young leaves would be helpful to understand the role of VOCs played in the host plant of ACP, which may be beneficial in designing ACP control strategies, as well as HLB detection. In this study, VOCs of HEAO, HLBO, HEAP, and HLBP were identified and analyzed from topographic plots by the HS-GC-IMS technique. The signal intensity of some VOCs in HLBO and HLBP showed a highly significant difference compared to those in HEAO and HEAP, respectively. HLB-infected leaves emitted more VOCs than healthy leaves. These findings were in accordance with the phenomenon where plants tend to increase VOC emissions after herbivore attack [32,33,34]. The PCA results clearly showed that HEAO and HLBO, as well as HEAP and HLBP, were in a relatively independent space and were well-distinguished. A novel method was developed to evaluate the characteristic VOCs of orange leaf samples by establishing the fingerprint with HS-GC-IMS and PCA. As well as we know, using HS-GC-IMS to analyze healthy and HLB-infected orange and pomelo young leaves has not been reported by other research groups. Taken together, information of VOCs identified by the HS-GC-IMS fingerprint and PCA could be a useful tool for the identification and classification of orange and pomelo leaf samples. Our study may help develop new strategies for the detection of HLB or find new attractants or repellents of ACP for prevention of HLB. It may also help explore plant–insect and plant–pathogen communication under biotic stresses. Unfortunately, many VOCs were not identified, due to the limited data library, especially for pomelo leaf samples. The development of a data library of HS-GC-IMS and more synergistic methods and approaches are expected for plant foliar VOC research in the future.

## Figures and Tables

**Table 1:** Headspace-gas chromatography ion mobility spectrometry (HS-GC-IMS) data of volatile organic compounds from healthy and HLB-infected orange leaves (HEAO and HLBO).

NO.	Compound	RI ^a^	Rt ^b^ (s)	Dt ^c^ (RIP ^d^ Relative)	Signal Intensity(HEAO)	Signal Intensity(HLBO)	*P* Value	Difference
1	Acetone	527.0	116.749	1.1172	2181.6 ± 48.5	4403.7 ± 96.3	<0.001	−101.9%
2	2-Butanone	604.3	145.839	1.0579	731.7 ± 89.3	481.9 ± 12.0	0.009	34.1%
3	2-Butanone dimer	601.6	144.824	1.2477	455.2 ± 91.4	459.9 ± 34.8	0.937	−1.0%
4	3-Pentanone	702.1	195.225	1.1063	1873.4 ± 100.9	1129.3 ± 24.6	<0.001	39.7%
5	3-Pentanone dimer	700.2	193.872	1.3535	4201.6 ± 131.5	1307.0 ± 55.1	<0.001	68.9%
6	Hexanal	790.6	273.363	1.2527	259.4 ± 43.1	79.7 ± 11.7	0.002	69.3%
7	Hexanal dimer	790.7	273.385	1.5642	51.5 ± 15.4	24.3 ± 1.9	0.039	52.8%
8	2-Hexenol	850.6	336.576	1.1795	947.0 ± 99.2	288.1 ± 70.8	0.001	69.6%
9	2-Hexenol dimer	850.2	336.033	1.5212	158.5 ± 45.2	24.4 ± 4.9	0.035	84.6%
10	Ethyl 2-methylbutanoate	847.7	333.085	1.2394	32.1 ± 7.2	228.1 ± 18.8	<0.001	−611.6%
11	Ethyl 2-methylbutanoate dimer	848.5	334.014	1.6553	25.7 ± 7.4	123.5 ± 21.3	0.002	−380.3%
12	(*Z*)−3-Hexen-1-ol	856.1	343.25	1.2314	189.0 ± 33.3	285.5 ± 37.3	0.029	−51.1%
13	(*Z*)-3-Hexen-1-ol dimer	856.1	343.204	1.512	531.2 ± 247.8	1457.6 ± 308.6	0.015	−174.4%
14	Limonene	1028.8	648.135	1.2181	2292.8 ± 3.8	2861.4 ± 73.7	<0.001	−24.8%
15	Limonene polymer	1031.0	652.017	1.2961	2319.9 ± 190.8	1650.0 ± 65.9	0.005	28.9%
16	Limonene polymer	1031.0	652.017	1.6592	1170.7 ± 41.8	1841.1 ± 230.1	0.008	−57.3%
17	Limonene polymer	1029.9	650.076	1.7295	1296.3 ± 115.4	1976.2 ± 276.4	0.017	−52.5%
18	α-Pinene	929.7	458.139	1.2185	3590.8 ± 44.2	4499.7 ± 133.8	<0.001	−25.3%
19	α-Pinene polymer	930.0	458.63	1.2923	698.4 ± 12.3	1144.4 ± 70.0	0.007	−63.9%
20	α-Pinene polymer	927.9	454.705	1.6744	1947.1 ± 150.8	5740.3 ± 1061.1	0.004	−194.8%
21	α-Pinene polymer	930.2	459.12	1.7323	269.3 ± 31.1	808.4 ± 137.0	0.003	−200.2%
22	Ethyl acetate	615.6	150.24	1.0973	292.9 ± 39.2	1091.6 ± 26.0	<0.001	−272.7%
23	Ethyl acetate dimer	615.6	150.24	1.3355	105.2 ± 19.4	6139.0 ± 49.9	<0.001	−5733.5%
24	Ethyl propanoate	711.9	202.517	1.1434	69.9 ± 6.4	220.5 ± 28.1	0.001	−215.7%
25	Ethyl propanoate dimer	710.6	201.527	1.4548	14.3 ± 0.8	59.8 ± 22.6	0.073	−317.6%
26	Ethyl 2-methylpropanoate	754.3	238.813	1.1905	45.9 ± 9.3	172.1 ± 4.6	<0.001	−274.8%
27	Ethyl 2-methylpropanoate dimer	752.1	236.833	1.5619	11.6 ± 0.2	58.4 ± 5.6	0.005	−402.8%
28	Benzaldehyde	953.0	503.079	1.1434	608.3 ± 14.2	427.2 ± 21.4	<0.001	29.8%
29	Benzaldehyde dimer	953.0	503.193	1.47	101.6 ± 2.3	85.2 ± 10.3	0.104	16.2%
30	3-Methylbutanol	734.6	221.157	1.2418	32.4 ± 3.4	254.5 ± 23.0	0.003	−684.9%
31	3-Methylbutanol dimer	734.1	220.7	1.4927	18.5 ± 2.8	72.1 ± 11.1	0.001	−289.1%
32	Methyl 2-methylbutanoate	770.0	253.595	1.1895	59.6 ± 3.9	157.8 ± 13.0	<0.001	−164.7%
33	Methyl 2-methylbutanoate dimer	770.0	253.595	1.5347	21.5 ± 1.0	49.5 ± 7.8	0.004	−129.8%
34	β-Ocimene	1052.9	690.729	1.2133	3753.4 ± 75.8	3654.0 ± 51.4	0.133	2.6%
35	β-Ocimene polymer	1051.9	688.902	1.2566	1533.9 ± 146.8	1386.7 ± 103.9	0.229	9.6%
36	β-Ocimene polymer	1052.4	689.815	1.6976	3809.0 ± 1056.0	7601.4 ± 113.5	0.003	−99.6%

RI ^a^: Retention index; Rt ^b^: Retention time; Dt ^c^: Drift time (RIP relative); RIP ^d^: Reactant ion peak. Values of signal intensity are given as mean ± SD (n = 3). *P* < 0.05: Significant, *P* < 0.01: Very significant, and *P* < 0.001: Highly significant.

**Table 2:** HS-GC-IMS data of volatile organic compounds from healthy and HLB-infected pomelo leaves (HEAP and HLBP).

NO.	Compound	RI^a^	Rt ^b^ (s)	Dt ^c^ (RIP ^d^ Relative)	Signal Intensity(HEAP)	Signal Intensity(HLBP)	*P* Value	Difference
1	2-Butanone	617.4	150.906	1.0595	1220.5 ± 47.5	1307.7 ± 106.4	0.265	−7.1%
2	2-Butanone dimer	616.7	150.640	1.2515	1404.5 ± 131.9	1589.0 ± 108.2	0.135	−13.1%
3	3-Pentanone	712.0	202.578	1.1028	1022.3 ± 35.8	259.0 ± 29.2	<0.001	74.7%
4	3-Pentanone dimer	710.2	201.249	1.3599	648.2 ± 51.1	51.6 ± 8.3	<0.001	92.0%
5	Acetone	548.7	124.904	1.1203	1575.1 ± 83.2	1593.1 ± 45.3	0.759	−1.1%
6	hexanal	800.3	282.722	1.2521	38.8 ± 4.0	32.0 ± 3.7	0.093	17.5%
7	Limonene	1036.5	661.717	1.2227	1584.6 ± 401.6	2620.9 ± 103.3	0.012	−65.4%
8	Limonene polymer	1037.0	662.589	1.2929	1997.1 ± 588.6	3978.6 ± 120.2	0.005	−99.2%
9	Limonene polymer	1036.5	661.717	1.6643	242.3 ± 116.4	929.6 ± 60.4	0.001	−283.7%
10	Limonene polymer	1036.0	660.845	1.7345	291.5 ± 103.3	990.3 ± 50.2	<0.001	−239.7%

RI ^a^: Retention index; Rt ^b^: Retention time; Dt ^c^: Drift time (RIP relative); RIP ^d^: Reactant ion peak. Values of signal intensity are given as mean ± SD (n = 3). *P* < 0.05: Significant, *P* < 0.01: Very significant, and *P* < 0.001: Highly significant.
